# Paeoniflorin ameliorates ulcerative colitis by modulating the dendritic cell-mediated T_H_17/T_reg_ balance

**DOI:** 10.1007/s10787-020-00722-6

**Published:** 2020-05-29

**Authors:** Kai Zheng, Jia Jia, Shihai Yan, Hong Shen, Ping Zhu, Jiangyi Yu

**Affiliations:** 1 Department of Gastroenterology, Affiliated Hospital of Nanjing University of Chinese Medicine, Nanjing, Jiangsu China; 2 Department of Endocrinology, Affiliated Hospital of Nanjing University of Chinese Medicine, Nanjing, Jiangsu China; 3 Laboratory of Pharmacology, Affiliated Hospital of Nanjing University of Chinese Medicine, Nanjing, Jiangsu China; 4 Department of Colon and Rectal Surgery, Affiliated Hospital of Nanjing University of Chinese Medicine, Nanjing, Jiangsu China

**Keywords:** Ulcerative colitis, Paeoniflorin, Dendritic cells, T_reg_, T_H_17, Immunological tolerance

## Abstract

Immunological tolerance is critical for maintaining gut homeostasis. An imbalance between interleukin-17 (IL-17)-producing T helper 17 (T_H_17) cells and regulatory T cells (T_reg_ cells) is involved in ulcerative colitis (UC) pathogenesis. Dendritic cells (DCs) are able to induce T cell differentiation. Paeoniflorin (PF) is a monoterpene glucoside that is commonly used for treatment of autoimmune disease. However, the immunological mechanism of PF involvement in UC treatment is unclear. The present study aimed to explore whether PF can restore the T_H_17/T_reg_ balance by modulating DCs. The effects of PF on DCs, T_H_17 cells and T_reg_ cells were measured. Furthermore, PF-treated DCs were injected into mice with 2,4,6-trinitrobenzenesulfonic acid (TNBS)-induced colitis. PF inhibited MHC-II and CD86 expression on the DC surface (*P* < 0.05), decreased interleukin (IL)-12 secretion in vitro and in vivo (*P* < 0.05), and restored the T_H_17/T_reg_ ratio in the mouse model of colitis (*P* < 0.05). PF-treated DCs diminished T_H_17 differentiation (4.26% in vitro and 1.64% in vivo) and decreased IL-17 expression (*P* < 0.05) while inducing CD4^+^CD25^+^Foxp3^+^ T_reg_ differentiation (7.82% in vitro and 6.85% in vivo) and increasing Foxp3 and IL-10 production (*P* < 0.05). Additionally, both PF and PF-treated DCs improved colonic histopathology in the mouse model of colitis (*P* < 0.05). In conclusion this study suggested that PF can ameliorate TNBS-induced colitis by modulating the DC-mediated T_H_17/T_reg_ balance.

## Introduction

Ulcerative colitis (UC) is a chronic inflammatory bowel disease (IBD) of unknown aetiology that affects the colon. The main UC symptoms include diarrhoea, rectal bleeding and abdominal pain. Systemic features, such as fever, fatigue and weight loss, are relatively common if all or most of the colon is involved in UC (Yu and Rodriguez 2017). UC has a higher prevalence in the Western hemisphere, but recently both the incidence and prevalence rates have been rising in the rest of the world, particularly in Asia (Ng et al. 2018). Although many current hypotheses implicate a combination of environmental, genetic and immunoregulatory factors, the cause of IBD remains unclear (Goethel et al. 2018).

The molecular mechanisms of IBD are complex, and it is widely accepted that autoreactive T helper (T_H_) cells are critically involved in the development of this disease (Cosmi et al. 2014; Hagihara et al. 2019). UC has historically been considered a T_H_2-driven disease (Ungaro et al. 2017). However, the importance of interleukin-17 (IL-17)-producing T helper (T_H_17) cells in the progression of UC has been recognized (Ueno et al. 2018). T_H_17 cells, a subset of CD4^+^ T effector cells, produce mainly IL-17 and have a proinflammatory function in the inflammatory response. Regulatory T cells (T_reg_ cells) are a special subset of T cells that have an immunoregulatory function and promote immunological tolerance. A hallmark of both natural and induced T_reg_ cells is the expression of the transcription factor Forkhead box P3 (Foxp3); patients with UC have a relatively low frequency of CD4^+^CD25^+^CD127^low^Foxp3^+^ T_reg_ cells (Mohammadnia-Afrouzi et al. 2015). Recent studies have suggested that the loss of homeostasis between T_H_17 cells and T_reg_ cells leads to the aberrant immune response of IBD (Britton et al. 2019; Shouval et al. 2017).

Dendritic cells (DCs), which are professional antigen-presenting cells, play pivotal roles in maintaining gut immune homeostasis and tolerance (Rutella and Locatelli 2011). Most DCs circulate in the body in an immature state, and intestinal DCs that present innocuous environmental antigens to naive T cells in the mesenteric lymph node (MLN) induce CD4^+^CD25^+^Foxp3^+^ T_reg_ cells differentiation (Huang et al. 2010). However, mature DCs can reduce T_reg_ cells differentiation (Lu et al. 2016).

**Fig. 1:**
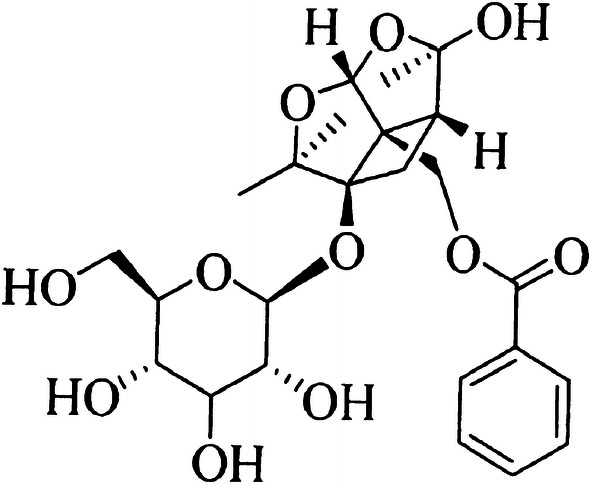
Chemical structure of PF

Paeoniflorin (PF) is a major bioactive monoterpene glucoside (C_23_H_28_O_11_, Fig. 1) extracted from the roots of *Paeonia lactiflora* (Wu et al. 2010), which is a herbal medicine that has been widely used to treat autoimmune diseases such as rheumatoid arthritis, IBD and type 1 diabetes in China (Zheng et al. 2017). Although few studies have reported on the anti-inflammatory activities of PF in immune diseases (Ko et al. 2018; Tu et al. 2019), the immune-mediated anti-inflammatory therapeutic mechanisms underlying the clinical effects of PF on UC have not been sufficiently investigated. Therefore, we explored the efficacy of PF in an experimental colitis model and elucidated its influence on T cells and DCs. The present study selected the well‑established model of 2,4,6-trinitrobenzenesulfonic acid (TNBS)‑induced experimental colitis, which is characterized by histological changes that manifest as multifocal areas of ulcers with a mild to severe inflammatory infiltrate, to evaluate the effect of PF (Catana et al. 2018). The present study might support the clinical application of PF for UC treatment.

## Materials and methods

### Mice and reagents

Male C57BL/6 mice (6–8 weeks old) were obtained from the Sippr-BK Laboratory Animal Co., Ltd. (Shanghai, China). Mice were housed in an animal facility under specific pathogen-free (SPF) conditions. All experimental procedures were approved by the ethics committee of the Affiliated Hospital of Nanjing University of Chinese Medicine. PF with a purity of 98% was purchased from Xiya Reagent Co., Ltd. (Shandong, China) and dissolved in sterile phosphate-buffered saline.

### DCs generation

Bone marrow-derived DCs were generated from C57BL/6 mouse bone marrow as described previously (Liu et al. 2014). Bone marrow was isolated, and red blood cells were lysed with 0.87% ammonium chloride buffer. The bone marrow cells (1 × 10^6^/ml) were suspended in complete RPMI-1640 medium with 20 ng/ml GM-CSF and 10 ng/ml IL-4 (ProSpec, Ness-Ziona, Israel) for 48 h to generate immature DCs (imDCs) and then divided into four groups: the imDC control group, PF-treated imDC group, LPS-treated imDC group and PF + LPS-treated imDC group. PF (50 μg/ml) was used to treat imDCs stimulated with 1 μg/ml LPS (Sigma‑Aldrich, Hamburg, Germany), and 36 h later, the supernatants were collected.

### Coculturing naive CD4^+^ T cells with DCs

Naive CD4^+^ T cells were isolated from C57BL/6 mouse splenocyte suspensions (1 × 10^7^ per well) by magnetic beads following the manufacturer’s instructions (Miltenyi Biotec, Auburn, CA, USA), and flow cytometry was employed to assess purity. There were five groups of T cells examined: the normal control group, imDC group, LPS-treated DC group, PF-treated DC group and PF-treated LPS-DC group. Naive CD4^+^ T cells were cocultured at a density of 1 × 10^6^ cells/ml with imDCs, LPS-treated DCs, PF-treated DCs or PF-treated LPS-DCs and incubated at 37 ℃ in a 5% CO_2_ atmosphere for 5 days.

### Experimental colitis induction in mice

TNBS was dissolved in alcohol and saline (50:50, v/v) to produce a stock solution. For colitis induction in C57BL/6 mice, TNBS (100 mg/kg body weight) was injected intrarectally using a 3.5F catheter (Wirtz et al. 2017). Forty-eight mice were divided into eight groups: a normal control group, a TNBS model group, a sulfasalazine (SASP) (Sine Pharmaceutical Co., Ltd., Shanghai, China) group, a prednisone (Aladdin Industrial Corporation, Shanghai, China) group, PF (5, 10 and 20 mg/kg) groups and a PF-treated DC group. The normal control group and TNBS model group received an intrarectal 0.9% saline injection at a dose of 10 ml/kg. PF was given via gastric gavage at doses of 5, 10 or 20 mg/kg/day; SASP was administered via gastric gavage at a dose of 100 mg/kg/day; and prednisone was given via gastric gavage at a dose of 3 mg/kg/day. Mice in the PF-treated DC group were injected with 1 ml (1 × 10^6^ cells/ml) of PF-treated DCs. All mice were treated for 7 days.

### Histological analysis

The obtained colonic samples were fixed in 4% neutral buffered formalin and embedded in paraffin to produce paraffin blocks. Each block was sliced into 4-μm-thick sections and stained with haematoxylin and eosin (H&E) according to standard protocols. The degree of histological damage was scored by two pathologists in a blinded manner and assessed according to a previously established standard, as follows (Scheiffele and Fuss 2002): 0, no evidence of inflammation; 1, low level of inflammation, with scattered infiltrating mononuclear cells (1–2 foci); 2, moderate inflammation, with multiple foci; 3, high level of inflammation, with increased vascular density and marked wall thickening; and 4, maximal severity of inflammation, with transmural leukocyte infiltration and loss of goblet cells.

### Immunohistochemistry

Fixed colonic samples were cut into 4-μm-thick sections, deparaffinized and rehydrated through a series of xylene and ethanol washes. Sections were blocked with 1% goat serum albumin for 10 min at room temperature and incubated overnight at 4 ℃ with an CD11c primary antibody (Abcam, Cambridge, UK). Then, a horseradish peroxidase‑conjugated secondary antibody was added and incubated for 60 min at 37 ℃. The sections were then dehydrated using an alcohol gradient, cleared in xylene and mounted for microscopic examination (DMI5000M; Leica, Solms, Germany) to count the number of positive cells.

### Flow cytometry analysis

Bone marrow-derived DCs were generated from C57BL/6 mouse bone marrow and then incubated with CD45 (PE), MHC-II (FITC) and CD86 (APC) primary antibodies. The PE-CD45 antibody was used to obtain DCs. DCs were phenotyped for MHC-II (FITC) and CD86 (APC) expression with a FACSCalibur™ flow cytometer using CellQuest software (Becton, Dickinson and Company, Fullerton, CA, USA). MLNs were processed to isolate mononuclear cells, which were incubated with anti-CD4 (FITC) and anti-CD25 (APC) primary antibodies for 20 min at 4 ℃, washed twice and fixed. The cells were subsequently stained with anti-Foxp3 PE-conjugated or anti-IL-17A PE-conjugated antibodies and analysed by FACS for T_reg_ cells or T_H_17 cells, respectively.

### Enzyme-linked immunosorbent assay (ELISA)

Samples were homogenized in 1 ml of ice-cold radioimmunoprecipitation assay (RIPA) lysis buffer containing 1% phosphatase inhibitor cocktail and 1% protease inhibitor cocktail. The lysate was centrifuged for 10 min, and the supernatant was transferred to 96-well ELISA plates. The IL-12, IL-10 and IL-17 levels in the cell suspension, MLN and colonic tissue samples were assayed with ELISA kits (Abcam, Cambridge, UK) according to the manufacturer’s instructions. The absorbance was measured spectrophotometrically at 450 nm.

### Quantitative real-time polymerase chain reaction (qRT‑PCR)

The IL-12, IL-10, IL-17 and Foxp3 transcript levels were evaluated by qRT-PCR. In brief, total RNA was isolated using TRIzol reagent (Invitrogen, Carlsbad, CA, USA) and reverse transcribed into cDNA using a PrimeScript RT reagent Kit with gDNA Eraser (TaKaRa Biotechnology, Dalian, China) according to the manufacturer’s instructions. Then, qRT-PCR was performed in 20-μl reaction mixtures containing SYBR Green Realtime PCR Master Mix (TOYOBO, Osaka, Japan), cDNA and 0.2 mmol/l each primer at 95 ℃ for 10 min, followed by 40 cycles of 95 ℃ for 5 s and 60 ℃ for 60 s. The data were collected using a StepOnePlus real-time PCR instrument (Applied Biosystems, Foster City, CA, USA). We normalized the results for each individual gene using the results for the housekeeping gene β-actin. The 2^− ΔΔCt^ method was used to calculate relative gene expression levels. The sequences of the PCR primer pairs (produced by Sangon Biotechnology, Shanghai, China) are the following (5′-3′): IL-12, GCTCGCAGCAAAGCAAGGTAA and CCATGAGTGGAGACACCAGCA; IL-10, AGGATGCACATCAAAAGGCTT and GGCCTCGGTTAGGAAGGATAC; IL-17, AGCACACCCGTCTTCTCTC and GCTGGAGTTCGCACTGTCC; Foxp3, CCCATCCCCAGGAGTCTTG and ACCATGACTAGGGGCACTGTA; and β-actin, CATCCGTAAAGACCTCTATGCCAAC and ATGGAGCCACCGATCCACA.

### Western blot analysis

Lysates were separated on 10% SDS-PAGE gels and then transferred to polyvinylidene fluoride membranes. The membranes were blocked with 5% defatted milk in Tris-buffered saline for 2 h at room temperature, followed by an overnight incubation at 4 ℃ with a primary anti-Foxp3 antibody (Abcam, Cambridge, UK) at a 1:1000 dilution. After three washes, the membranes were probed with an HRP-conjugated secondary antibody (1:5000) for 2 h at room temperature, and the visualized protein bands were detected with an enhanced chemiluminescence (ECL) detection system (Santa Cruz Biotechnology, Santa Cruz, CA, USA). Normalization was performed against β-actin expression.

### Statistical analysis

The data are shown as the mean ± standard deviation. Statistically significant differences among groups were determined by one-way ANOVA followed by Tukey's multiple comparisons test (GraphPad Prism 6.0, GraphPad Software Inc., San Diego, CA, USA). Differences were considered significant when the *P* value was less than 0.05.

## Results

### PF inhibited DC maturation in vitro

**Fig. 2:**
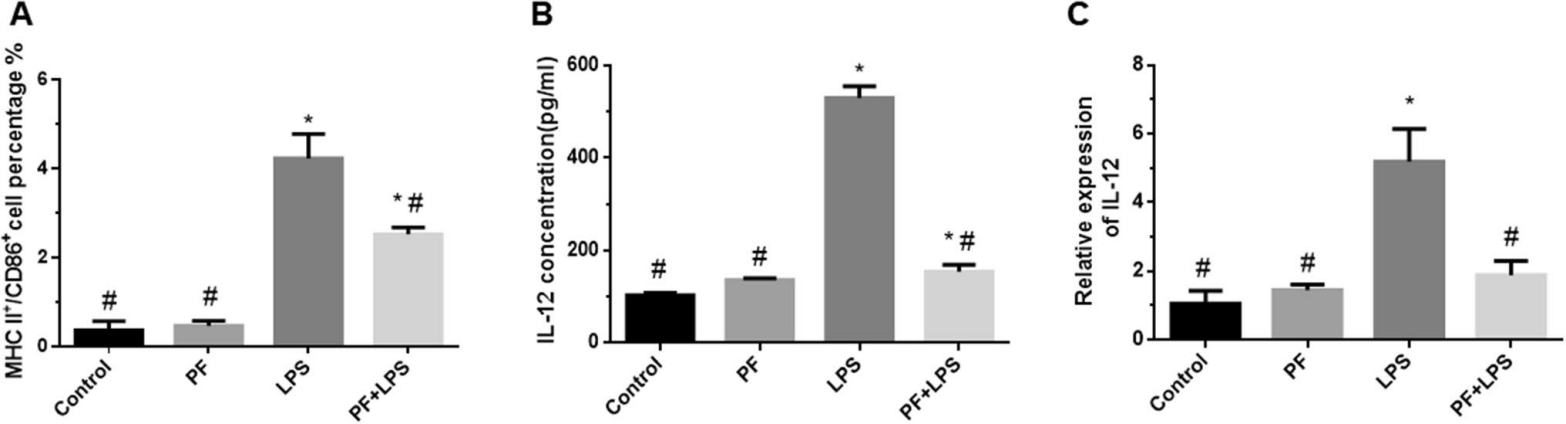
PF inhibited DC maturation in vitro. Bone marrow-derived DCs were treated with medium alone, 50 μg/ml PF, 1 μg/ml LPS, or 50 μg/ml PF and 1 μg/ml LPS for 36 h. **a** After 36 h, the supernatants were collected. Surface MHC-II and CD86 expression was assessed by flow cytometry. **b**, **c** The level of the proinflammatory cytokine IL-12 in supernatants was measured by ELISA and qRT‑PCR. *P < 0.05 compared with the control group, ^#^*P* < 0.05 compared with the LPS group

MHC-II and the costimulatory molecule CD86 are expressed on the surface of DCs. Mature DCs express high levels of MHC-II and CD86. We defined positive cell-surface MHC-II and CD86 expression as an indicator of mature DCs. Bone marrow-derived DCs were generated from C57BL/6 mice and treated with medium alone, 50 μg/ml PF, 1 μg/ml LPS, or 50 μg/ml PF and 1 μg/ml LPS, and the supernatants were collected 36 h later. The MHC-II^+^CD86^+^ cells in the single-cell suspension represented mature DCs. The percentages of mature DCs in the LPS group and PF + LPS group were higher than that in the control group. In contrast, compared with that in the LPS group, the percentages of mature DCs in the PF group and in the PF + LPS group were significantly decreased (Fig. 2a).

Mature DCs produce high levels of the proinflammatory cytokine IL-12. To determine DC maturity, we tested the IL-12 expression levels using ELISA and qRT‑PCR. The LPS group had higher IL-12 protein and mRNA levels than the control and PF groups. Compared with the LPS group, the PF + LPS group had significantly decreased IL‑12 concentrations and mRNA levels (Fig. 2b, c), which showed that PF could interrupt the effects of LPS stimulation.

### PF-treated DCs modulated the T_H_17/T_reg_ balance in vitro

To investigate whether PF-treated DCs modulate T cell differentiation, we purified naive CD4^+^ T cells from mouse splenocytes and cocultured the cells with medium alone, imDCs, LPS-treated DCs, PF-treated DCs or PF-treated LPS-DCs.

**Fig. 3:**
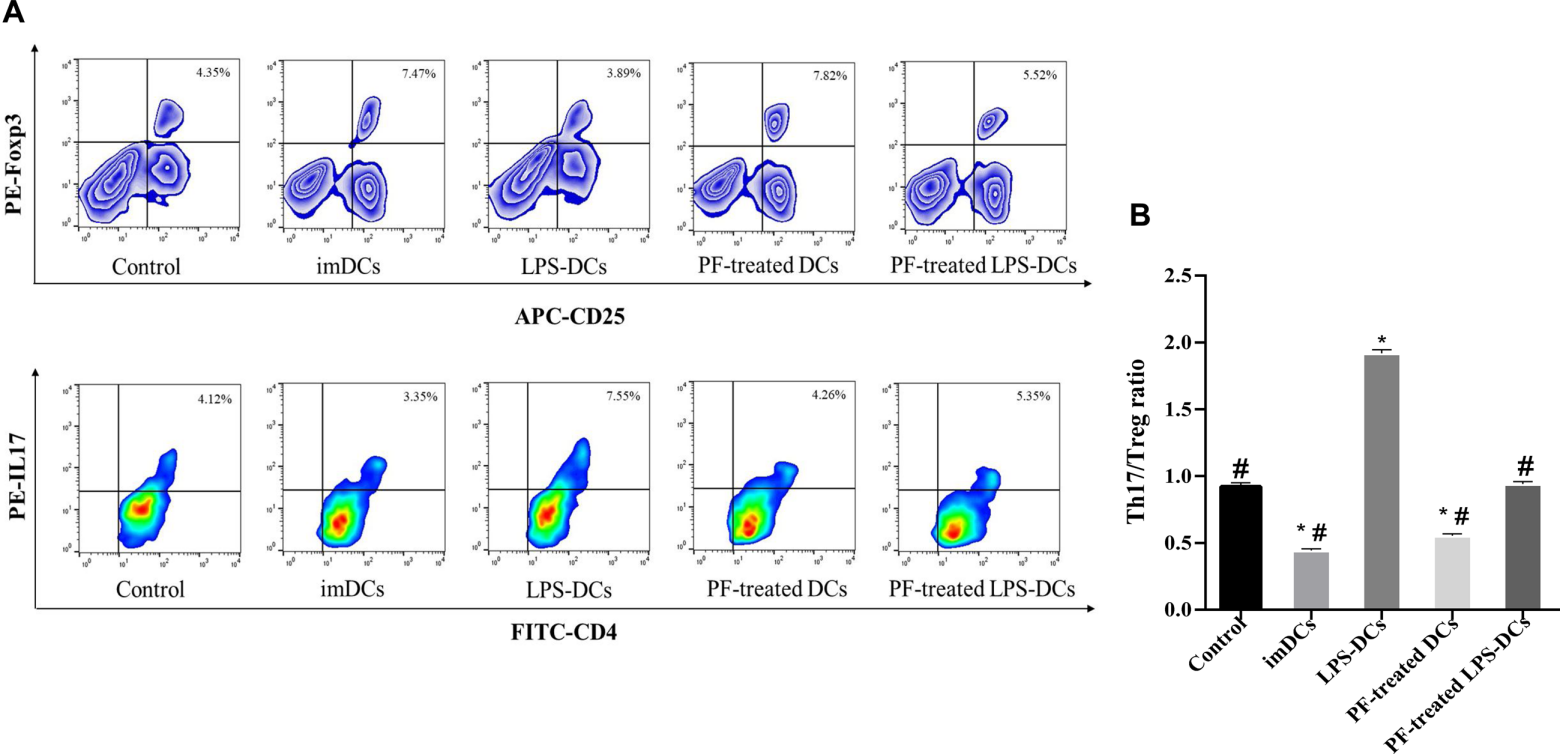
PF-treated DCs reduced the T_H_17/T_reg_ ratio. Naive CD4^+^ T cells were cocultured with medium alone, imDCs, LPS-treated DCs, PF-treated DCs or PF-treated LPS-DCs for 5 days. **a** After 5 days, the frequencies of T_reg_ cells and T_H_17 cells were analysed by flow cytometry. **b** The T_H_17/T_reg_ ratio showed that PF-treated DCs and imDCs modulated the T_H_17/T_reg_ balance. *P < 0.05 compared with the control group, ^#^*P* < 0.05 compared with the LPS-treated DC group

Figure 3a shows that the imDC and PF-treated DC groups exhibited significantly increased differentiation of naive CD4^+^ T cells into CD4^+^CD25^+^Foxp3^+^ T_reg_ cells and that the LPS-DC group had significantly increased differentiation of naive CD4^+^ T cells into T_H_17 cells, whereas T_H_17 cell accumulation decreased in the imDC, PF-treated DC and PF-treated LPS-DC groups compared with the LPS-DC group. The PF-treated LPS-DC, PF-treated DC and imDC groups also had significantly lower T_H_17/T_reg_ ratios than the LPS-treated DC group (Fig. 3b).

**Fig. 4:**
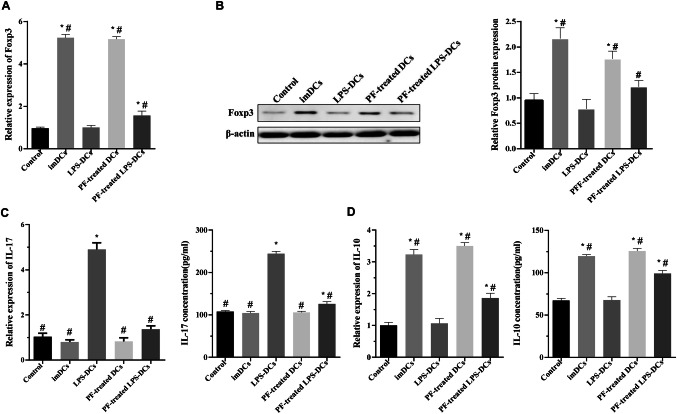
PF-treated DCs increased Foxp3 and IL-10 expression and decreased the IL-17 level in naive CD4^+^ T cells after 5 days of coculture. **a**, **b** Foxp3 mRNA and protein levels were detected by qRT‑PCR and western blot, respectively. **c**, **d** The IL-10 and IL-17 levels in the supernatants were measured by qRT‑PCR and ELISA. *P < 0.05 compared with the control group, ^#^*P* < 0.05 compared with the LPS-treated DC group

T_H_17 cells produce the proinflammatory cytokine IL-17, whereas T_reg_ cells produce the anti-inflammatory cytokine IL-10. To explore the influence of PF-treated DCs on T_reg_ cells and T_H_17 cells, we detected Foxp3, IL-10 and IL-17 expression by qRT‑PCR, western blot and ELISA. The results are shown in Fig. 4 and indicate that the PF-treated DCs and imDCs enhanced the Foxp3 mRNA and protein levels compared with those of the normal control group, and the PF-treated LPS-DCs showed significantly increased Foxp3 levels compared with those of the LPS-DC group (Fig. 4a, b). Furthermore, qRT‑PCR and ELISA analyses revealed that compared with the LPS-treated DCs, the PF-treated LPS-DCs, PF-treated DCs and imDCs exhibited significantly decreased IL-17 secretion and increased IL-10 secretion (Fig. 4c, d).

### PF and PF-treated DCs ameliorated TNBS-induced colitis in vivo

**Fig. 5:**
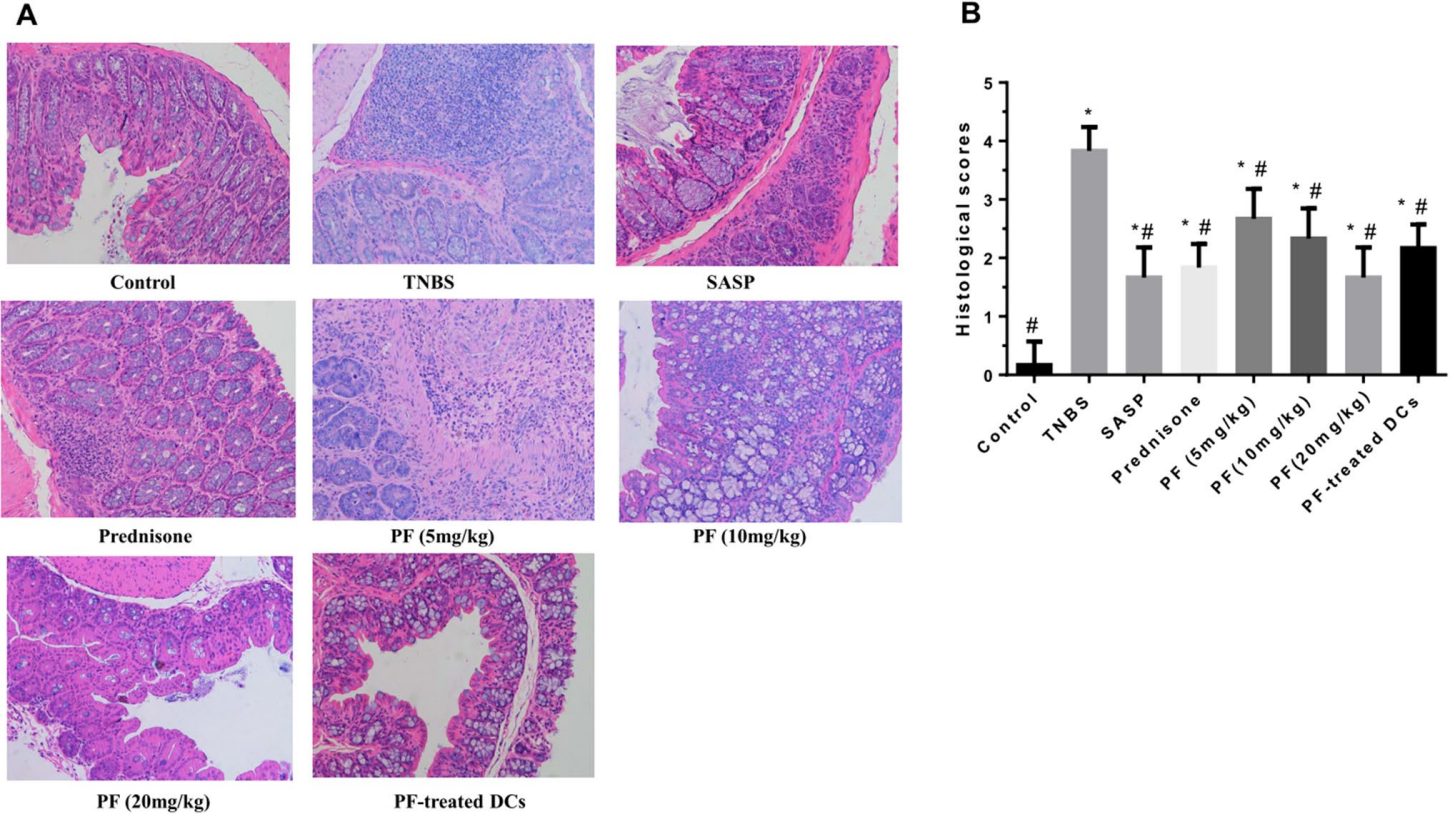
Histological improvements induced by PF or PF-treated DCs in TNBS-induced colitis mice. SASP (100 mg/kg/day), prednisone (3 mg/kg/day), PF (5, 10 and 20 mg/kg/day) or PF-treated DCs (1 × 10^6^ cells/day) were administered to TNBS-induced experimental colitis mice. After 7 days, mouse colons were isolated and harvested. **a** With H&E staining, histological changes were evaluated under a microscope (magnification, × 200). **b** Histopathological scores of colons from colitis mice are shown. Histological grading was determined as described in the “Materials and methods” section. *N* = 6 mice per group. *P < 0.05 compared with the control group, ^#^*P* < 0.05 compared with the TNBS group

Colons from TNBS-induced mice revealed striking hyperaemia and inflammation, whereas colons from control mice showed no macroscopic signs of inflammation. Histologically, compared with those from control mice, the colonic tissue samples from TNBS‑induced colitis mice exhibited a reduced number of goblet cells, crypt loss, infiltration by inflammatory cells and extensive mucosal layer destruction. Compared with the TNBS group, the PF group (20 mg/kg) and the PF-treated DC group showed progressive crypt architecture and goblet cell restoration and a reduction in inflammatory cell infiltration to a level similar to that in the SASP and prednisone group, with a significant decrease in histological scores (Fig. 5).

### PF inhibited DC maturation in TNBS-induced colitis

**Fig. 6:**
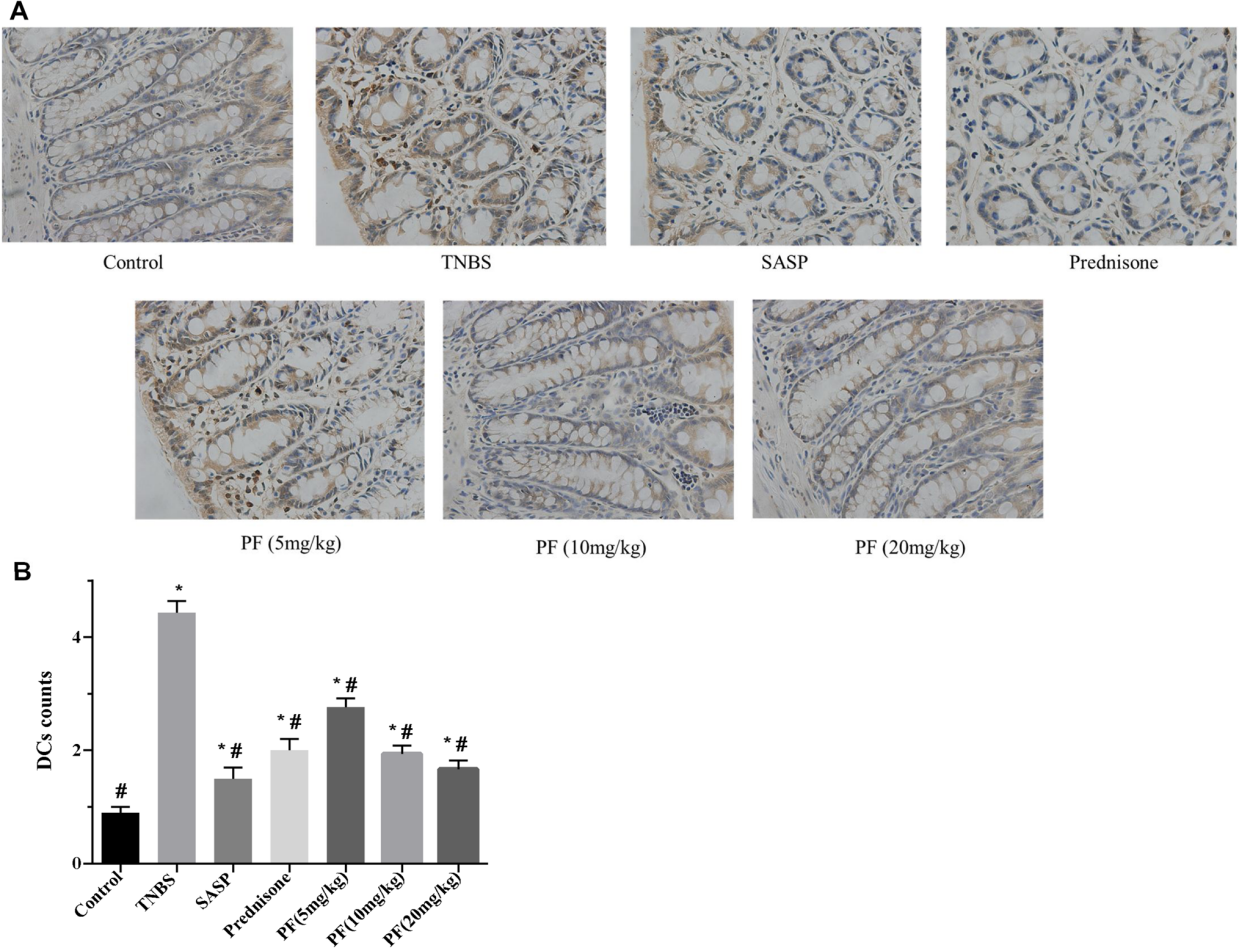
PF reduced DC counts in TNBS-induced colitis. Mouse colons were dissected after 7 days of treatment administration, and fixed colonic samples were cut into 4-μm-thick sections. **a** Immunostaining was performed with a mouse monoclonal antibody against CD11c. Representative immunohistochemical staining of colonic tissue samples shows mature DCs (brown) (magnification, × 400). **b** DC counts were calculated. *P < 0.05 compared with the control group, ^#^*P* < 0.05 compared with the TNBS group

Immunostaining with anti-mouse CD11c antibodies was performed on colon samples from C57BL/6 mice. CD11c^+^ DCs were sparse in the normal colon tissue of the control group and dense in the tissue of the TNBS-induced colitis group. The CD11c^+^ DC counts in the SASP group, prednisone group and PF (5, 10 and 20 mg/kg) groups were significantly lower than those in the TNBS-induced colitis group (Fig. 6).

**Fig. 7:**
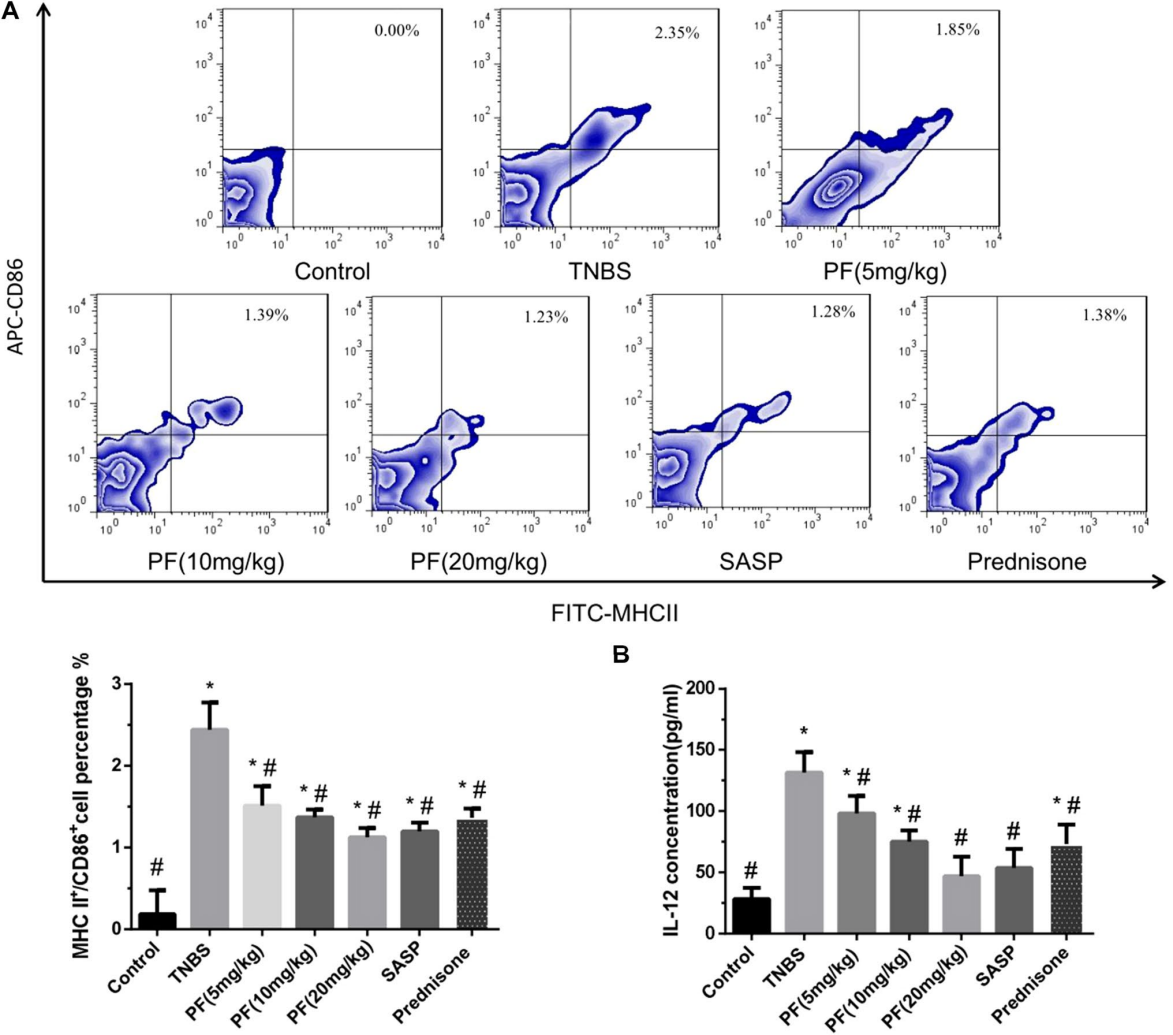
PF inhibited DC surface molecule expression and cytokine secretion in TNBS-induced colitis. The MLN was removed from mice 7 days after drug treatment. **a** The percentage of MHC-II^+^CD86^+^ DCs in the MLN was assessed in the CD45^+^ gate by flow cytometry. **b** The secretion of the cytokine IL-12 was measured by ELISA. *P < 0.05 compared with the control group, ^#^*P* < 0.05 compared with the TNBS group

We detected the percentage of MHC-II^+^CD86^+^ DCs in the MLN by FACS. As shown in Fig. 7a, the percentage of MHC-II^+^CD86^+^ DCs was significantly higher in TNBS-induced colitis mice than in control mice. In contrast, the percentage of mature DCs in the PF groups (5, 10 and 20 mg/kg), prednisone group and SASP group was lower than that in the TNBS-induced colitis group.

IL-12 production by mature DCs was increased in the TNBS-induced colitis model but decreased in response to PF, SASP or prednisone treatment and correlated with the PF dose (Fig. 7b).

### PF and PF-treated DCs modulated the T_H_17/T_reg_ balance in TNBS-induced colitis

**Fig. 8:**
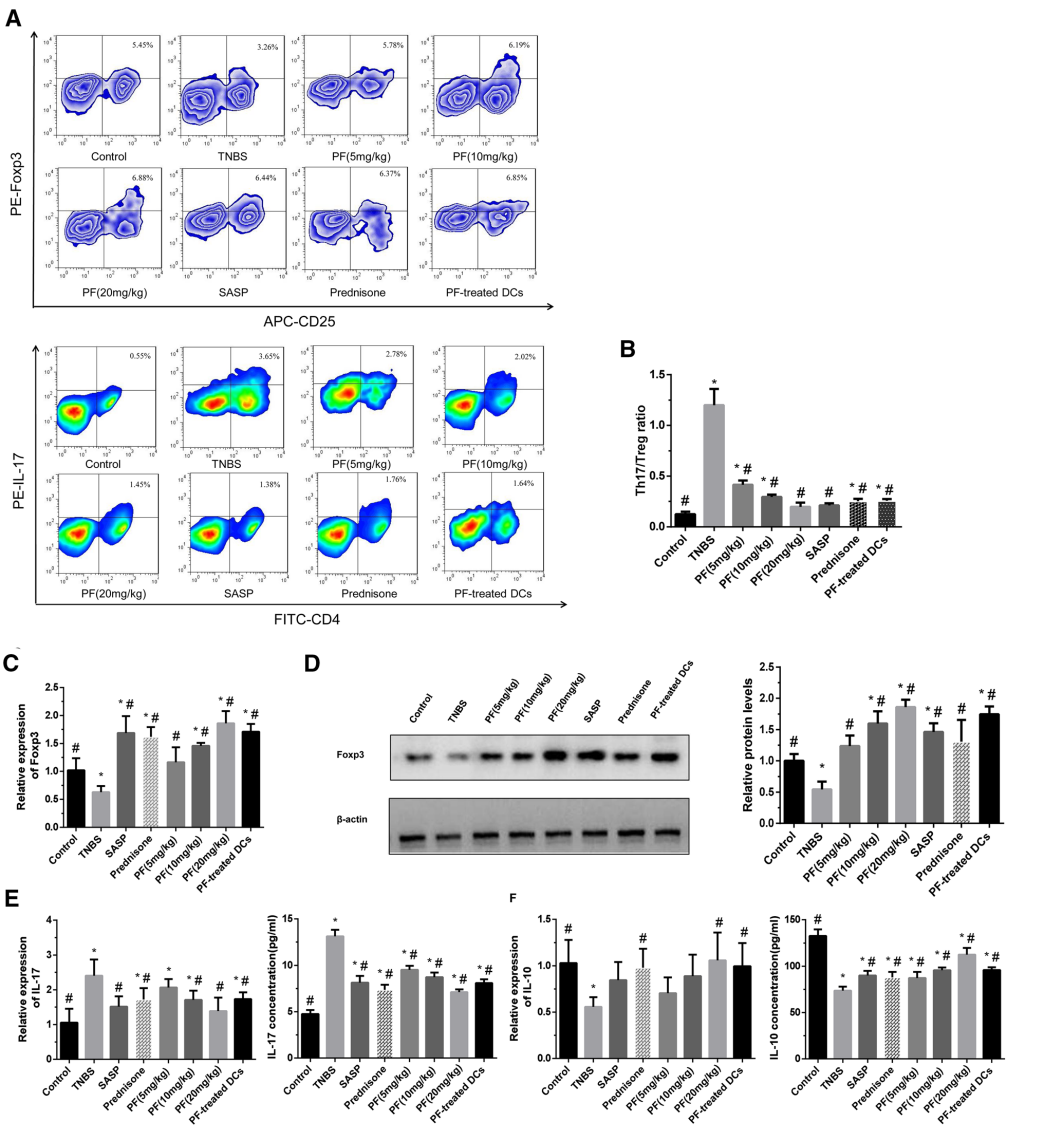
PF and PF-treated DCs reduced the T_H_17/T_reg_ ratio and T_H_17 cell‑associated cytokines and promoted T_reg_‑associated transcription factor and cytokine production in TNBS-induced colitis. After 7 days of treatment administration, the MLN and colons were dissected. **a** The frequencies of T_reg_ cells and T_H_17 cells in the MLN were analysed by flow cytometry. **b** PF (5, 10 and 20 mg/kg/day) reduced the T_H_17/T_reg_ ratio in a dose-dependent manner, with higher doses achieving greater decreases in the ratio. **c**, **d** The mRNA and protein levels of the T_reg_‑associated transcription factor Foxp3 in colonic tissue samples were detected by qRT‑PCR and western blot, respectively. **e**, **f** The levels of the T_H_17‑associated cytokine IL-17 and T_reg_‑associated cytokine IL-10 in colonic tissue samples were measured by qRT‑PCR and ELISA. *P < 0.05 compared with the control group, ^#^*P* < 0.05 compared with the TNBS group

To detect T cell differentiation, T_H_17 cells and T_reg_ cells in the MLN were enumerated by FACS. As shown in Fig. 8a, the percentage of CD4^+^CD25^+^Foxp3^+^ T_reg_ cells was markedly decreased in the TNBS-induced mice, whereas the administration of PF or PF-treated DCs markedly increased this percentage, as did treatment with SASP or prednisone. The percentage of T_H_17 cells was increased in the TNBS-induced colitis group compared with that in the control group. Administration of PF (5, 10 and 20 mg/kg), PF-treated DCs, SASP or prednisone lowered the percentage of T_H_17 cells in the TNBS-induced colitis model. Additionally, the percentage of T_H_17 cells was obviously reduced in a PF dose-dependent manner. The PF (5, 10 and 20 mg/kg), PF-treated DC, SASP and prednisone groups also showed significantly lower T_H_17/T_reg_ ratios than the TNBS-induced colitis group (Fig. 8b).

The results showed that Foxp3 mRNA and protein expression in colonic tissue samples was dramatically decreased in the TNBS-induced colitis model group but increased in the PF (5, 10 and 20 mg/kg), PF-treated DC, SASP and prednisone groups compared with the TNBS-induced colitis model group (Fig. 8c, d). We next assessed IL-17 and IL-10 secretion by qRT-PCR and ELISA. IL-17 levels in colonic tissue samples from the TNBS-induced colitis group were dramatically higher than those in samples from the control group. In contrast, IL-17 levels decreased after the administration of PF (10 and 20 mg/kg), PF-treated DCs, SASP or prednisone in the TNBS-induced colitis model (Fig. 8e). IL-10 levels were decreased in the TNBS-induced colitis group compared with the control group but increased after the administration of PF (20 mg/kg), PF-treated DCs or prednisone (Fig. 8f).

## Discussion

UC is a chronic inflammatory disease of unknown aetiology that affects the colon. The pathophysiology of UC has been extensively studied, and genetic and environmental factors and immune system dysregulation have been found to be involved (Goethel et al. 2018; Ordas et al. 2012). The fundamental treatment for UC is 5-aminosalicylic acid administration. SASP, which is a prodrug that is cleaved in the colon by bacteria to release 5-aminosalicylic acid and sulfapyridine, is useful in the treatment of active UC as well as in the prevention of relapses when the disease is in remission (Cooke 1969). Steroids are another effective therapy for inducing remission in patients with moderate and severely active UC.

In ancient Chinese medicine, the Yellow Emperor’s Canon of Internal Medicine (722 BC) described symptoms (abdominal pain, diarrhoea and rectal bleeding) of a disease resembling UC (Kirsner 2001). *Paeonia lactiflora* has been used for over 1000 years in Chinese medicine to treat this disease. As the principal bioactive component of *P. lactiflora*, PF was shown to exert anti-inflammatory effects on UC in previous studies; PF inhibits the MAPK/NF-κB signalling pathway and suppresses IL-2, IL-6, TNFα and IFNγ levels (Gu et al. 2017).

In this study, we demonstrated that PF, SASP and prednisone could downregulate IL-17, increase the IL-10 level and improve histological scores, which had a marked treatment effect on the TNBS-induced UC model. To further understand the mechanism by which PF exerts its immunoregulatory actions, we investigated the influence of PF on DCs and T cells. The results showed that PF decreased mature DC counts in the TNBS-induced colitis model and reduced the percentage of MHC-II^+^CD86^+^ DCs and the expression of IL-12 in vitro and in vivo, which means that PF can inhibit DC maturation from imDCs. PF significantly reduced the T_H_17/T_reg_ ratio in the TNBS-induced colitis model. Furthermore, to investigate whether PF regulates the T_H_17/T_reg_ ratio via imDCs, we injected PF-treated DCs into TNBS-induced colitis mice. Our data indicated that similar to PF, PF-treated DCs could lower histological scores and decrease the T_H_17/T_reg_ ratio. This study confirms that PF ameliorates UC by modulating the DC-mediated T_H_17/T_reg_ balance.

Gut immune responses are normally regulated to maintain mucosal immunological tolerance, which can be used to avoid inflammatory disease in experimental animal models of type 1 diabetes, arthritis and IBD (Mowat and Bain 2011). Immunological tolerance destruction is an important pathogenic process in UC (Yamada et al. 2016). The principal peripheral tolerance mechanisms are anergy, suppression by T_reg_ cells, and T_reg_ development induction instead of effector T cell development (Kalekar et al. 2016). There are at least two types of T_reg_ cells: natural T_reg_ cells and induced T_reg_ cells (Adeegbe and Nishikawa 2013). Natural T_reg_ cells develop in the thymus. Induced T_reg_ cells are differentiated in the periphery from naive T cells under low-dose antigenic stimulation, which is involved in immunological tolerance maintenance in the gastrointestinal tract. Foxp3 is a key factor in T_reg_ development. Both PF and PF-treated DCs could upregulate Foxp3 expression, prompting naive T cells to differentiate into T_reg_ cells in our study. T_H_17 cells are an effector T cell subset that can activate immune responses to destroy immunological tolerance. Loss of balance between T_reg_ cells and T_H_17 cells is thought to lead to UC development (Ueno et al. 2018). Our study showed that both PF and PF-treated DCs could lower the percentage of T_H_17 cells, reducing the T_H_17/T_reg_ ratio in vivo. The results revealed that PF and PF-treated DCs induced immunological tolerance. The excessive induction of cytokines, including IL-6 and IL-12, from DCs may support T_H_17 lymphocyte polarization and development. Our study showed that PF significantly decreased the IL‑12 concentrations and mRNA levels from DCs. The mechanism of PF-treated DCs decreasing the percentage of T_H_17 cells may involve the IL-12 and JAK/STAT pathway. STAT3 has an important role in T cell-mediated immunity, including the proliferation and migration of T cells, differentiation into T_H_17 cells and balance between T_reg_ and T_H_17 cells. Another member of the IL-12 superfamily, IL-23, is important for propagating T_H_17 responses. Blocking IL-12/IL-23p40 might suppress the differentiation of both Th1 and T_H_17 cells. Activated STAT3 regulates T_H_17 cell differentiation by participating in the transcriptional activation of several T_H_17 regulatory genes, including those encoding IL-23R and RORγt.

In the periphery, DCs play a critical tolerogenic role, extending the immune homeostasis maintenance and blocking autoimmune responses (Iberg et al. 2017). There is mounting evidence that DCs also establish and maintain immunological tolerance (Steinman et al. 2003). DCs within the intestinal mucosa directly sample the intestinal tract lumen and transport antigens to the MLN in a CCR7-dependent manner (Chieppa et al. 2006). In the MLN, antigen-laden DCs promote naive T cell differentiation into Foxp3^+^ T_reg_ cells to maintain immunological tolerance. The subsets of DCs that induce or enhance tolerance are called tolerogenic DCs (Maldonado and von Andrian 2010). Immunosuppressants frequently affect DC immunogenicity by interfering with DC maturation, forming pharmacologically induced tolerogenic DCs (Maldonado and von Andrian 2010). MHC-II, CD86 and CD11c are indicators of DC maturation. In our study, PF decreased MHC-II, CD86 and CD11c expression on the surface of the DCs, which indicated that PF can inhibit DC maturation, and PF-treated DCs increased naive T cell differentiation into Foxp3^+^ T_reg_ cells in vitro and in vivo, which revealed that PF-treated DCs were a type of tolerogenic DC. Tolerogenic DCs modulate T cell-mediated responses through a variety of mechanisms (Horton et al. 2017). Tolerogenic DCs may polarize naive T cells towards a regulatory phenotype through surface expression of the immunoregulatory molecule PD-L1. Some evidence suggests that the ligation of surface PD-L1 triggers IL-10 production, which consequently polarizes naive T cells into T_reg_ cells (Kuipers et al. 2006). However, another study identified Lkb1 as a regulatory switch in DCs for controlling T_reg_ homeostasis, the immune response and tolerance, and the number of T_reg_ cells was negatively regulated by the kinase Lkb1 in DCs (Chen et al. 2018). Therefore, the signalling pathway initiated by PF-treated DCs to promote Foxp3^+^ T_reg_ differentiation remains to be further elucidated.

IL-17 is a characteristic inflammatory cytokine expressed by T_H_17 cells. T_H_17 cells are a subpopulation of IL-17^+^ cells, and IL-17 acts as a trigger of proinflammatory responses at the inflammation site in the intestine. The anti-inflammatory cytokine IL-10 dampens intestinal inflammation. PF downregulated IL-17 and increased the IL-10 levels in this study, indicating that PF has anti-inflammatory properties. PF inhibited DC maturation, and PF-treated DCs could also decrease IL-17 and increase the IL-10 levels in experimental colitis, demonstrating that PF exerts its anti-inflammatory effects through immune regulation.

In summary, this study examined the effects of PF-treated DC intervention on naive T cells and a TNBS-induced colitis model, and the findings show that PF can ameliorate TNBS-induced colitis by modulating DCs to restore the T_H_17/T_reg_ balance. Our study provides insights into the role of PF as a unique therapeutic agent in UC treatment and illustrates the underlying anti-inflammatory mechanism of PF from an immunological perspective.
